# Factors Associated With Depressive Symptoms in Individuals Who Have Experienced COVID-19 Self-Quarantine

**DOI:** 10.3389/fpubh.2022.810475

**Published:** 2022-04-27

**Authors:** Hye-Young Jang, Young Ko, Song-Yi Han

**Affiliations:** ^1^School of Nursing, Hanyang University, Seoul, South Korea; ^2^College of Nursing, Gachon University, Incheon, South Korea; ^3^Department of Nursing Science, Sunmoon University, Asan-si, South Korea

**Keywords:** coronavirus, COVID-19, COVID-19 measures, depressive symptom, self-quarantine

## Abstract

The purpose of this study was to identify the factors associated with depressive symptoms in individuals who have experienced self-quarantine because of coronavirus disease exposure or infection using Lazarus and Folkman's stress, coping, and adaptation theory, and George's Social Antecedent Model of Depression. This was a cross-sectional study that used data from the 2020 Korean Community Health Survey. A complex sample design was used to analyze the data. Descriptive statistics, the Rao-Scott X^2^ test, and logistic regression analysis were conducted to identify factors associated with depressive symptoms. Approximately 5.3% of the subjects had depressive symptoms. The factors associated with depressive symptoms were age, level of education, household income, changes in daily life due to coronavirus disease, whether someone provided assistance during the self-quarantine, perceived health status, and hospital consultation due to depressive symptoms. The findings of this study will be utilized as basic data for the development of programs to alleviate and prevent depressive symptoms in self-quarantine individuals.

## Introduction

In March 2020, the World Health Organization declared coronavirus disease (COVID-19) a pandemic, in order to promote international cooperation and response. Many countries have established COVID-19 measures, such as social distancing and quarantining to prevent the spread of the disease (1).

In particular, proper management and control of individuals who are in contact with COVID-19 infected patients are of utmost importance to prevent the spread of COVID-19. In Korea and many other countries, infected patients and those who have been in close contact with infected patients are isolated for 2 weeks as a primary response (2–4). Individuals under self-quarantine are physically isolated and prohibited to make any direct contact with others and to share daily items with others for at least 14 days. Public health officers monitor them by the self-quarantine safety protection app (5).

Such physical isolation is effective in preventing the spread of COVID-19; however, self-quarantine measures not only limit the interactions of the quarantined individual, but also have negative economic, emotional, and social effects on him or her (6–8). Lee et al. (9) showed that individuals practicing self-quarantine are highly likely to experience fear and uncertainty about infection and psychological withdrawal. In addition, interruptions in their social relationships lead to a sense of loss, depression, anxiety, stress, and fear of stigmatization, and isolation of their family members further causes psychological pain such as guilt and depression (6–8). Therefore, it is important to minimize the negative consequences of self-quarantine on mental health.

Previous studies on self-quarantine and depression due to infectious diseases found that 31.2% of those in self-quarantine due to severe acute respiratory syndrome showed depressive symptoms, and 3.0% of individuals who were in contact with patients with Middle East respiratory syndrome showed depressive symptoms after self-quarantine. In addition, the incidence of depression has been found to be 2.5 times higher in those who have experienced self-quarantine than in those who have not (3, 10, 11). A study on adults in the United States indicated that the prevalence of depression was three-folds higher during the COVID-19 pandemic than before the pandemic (12). In Korea, in 2020, 22.8% of adults aged 19 years or older were at risk for depression, which was six times higher than the 3.8% reported for 2018, before the COVID-19 pandemic (13). These findings suggest that depression experienced by those in self-quarantine results from the stress of adapting to sudden environmental changes. As the prevalence of depression is high in those practicing self-quarantine, it is necessary to systematically analyze the relationship between risk factors and depression in this group. Therefore, in this study, we applied Lazarus and Folkman's (14) stress, coping, and adaptation theory and George's (15) Social Antecedent Model of Depression (SAMD) to identify the factors of depression in individuals who have experienced self-quarantine because of coronavirus disease exposure or infection. The SAMD includes biological, psychological, and social factors rather than the fragmentary aspects of the cause of depression (15) and is ideal for the systemic evaluation of various factors related to depression in those who were in self-quarantine due to COVID-19.

Therefore, we identified the risk and buffering factors for depression in those who were in self-quarantine due to COVID-19 and provided basic data to improve our understanding of depression in this group and to seek adequate measures for treatment and prevention.

### Conceptual Framework

**Figure 1:**
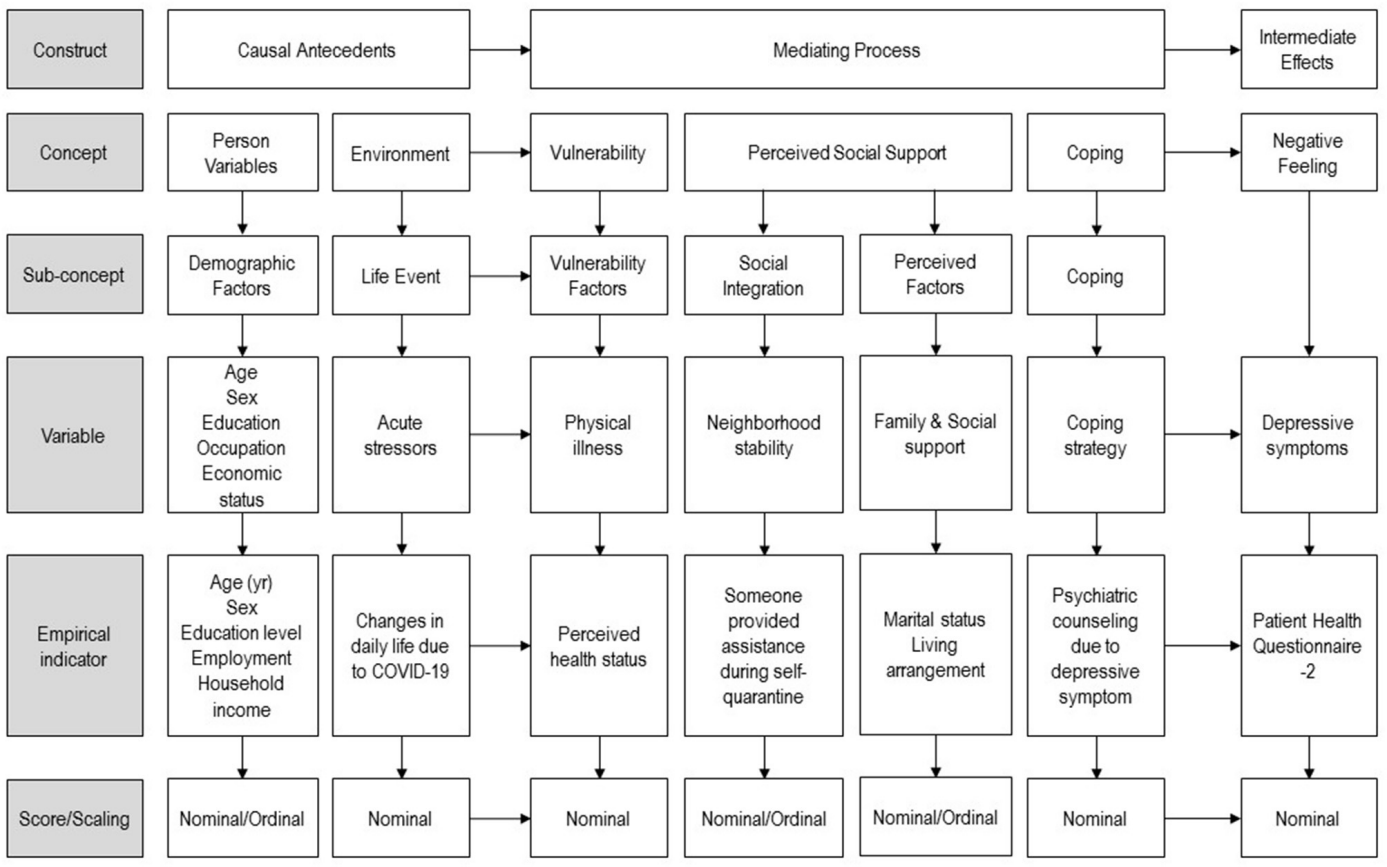
Substruction model of the theory of this study.

To identify the factors of depression in subjects who had experienced self-quarantine during COVID-19, Lazarus and Folkman's (14) stress, coping, and adaptation theory, and George's (15) SAMD were used to establish the conceptual framework of the study (Figure 1). Lazarus and Folkman's (14) theory has been used as a theoretical framework in many previous studies on stress, coping, and adaptation by systematically and logically explaining the overall process of evaluation, coping, and adaptation and causal antecedents of stressful events. In addition, George's (15) theory explains the relationship between depression and various factors at different stages to systematically and comprehensively measure the factors affecting depression. The SAMD has six stages: (1) demographic factors; (2) early life events and achievements; (3) later life events and achievements; (4) social integration; (5) vulnerability and protective factors; and (6) provoking and coping efforts.

The conceptual framework used in this study was constructed by modifying the factors of each stage of the SAMD to consider the situational characteristics of self-isolated individuals during the COVID-19 pandemic. Contextual factors included general characteristics of the subjects (age, gender, education, occupation, economic status) and recent events (changes in daily life due to COVID-19). Factors related to individual cognition and coping included social integration (whether someone provided assistance during the self-quarantine), vulnerability factors (perceived health status), protective factors (marital status, living arrangement), and coping factors (hospital consultation due to depressive symptom). The negative outcome variable was depression.

## Materials and Methods

### Research Design

This was a cross-sectional study that used data from the 2020 Korean Community Health Survey (KCHS) to identify factors associated with depressive symptoms among individuals who had experienced self-quarantine during the COVID-19 pandemic using Lazarus and Folkman's (14) stress, coping, and adaptation theory and George's (15) SAMD.

### Participants and Data

This study analyzed data from the KCHS. Since 2017, the Research Ethics Review Committee (RERC) of the Centers for Disease Control and Prevention decided that the KCHS corresponds to a study conducted by the state for public welfare. Therefore, on the basis of the opinion that it is possible to conduct an investigation without the approval of the RERC, data were collected without review by the RERC. Written informed consent was obtained from all subjects before participation. The data were collected in accordance with the disclosure and management regulations of the Korea Disease Control and Prevention Agency. We conducted this study with the approval of the institutional review board of the Gachon University to which the researchers belong (No. 1044396-202109-HR-198-01).

The KCHS has been conducted annually since 2008 by the Korea Disease Control and Prevention Agency to provide population-based statistics for developing and evaluating national healthcare plans. The KCHS is a nationwide, community-based health survey and the target population is adults aged 19 years or older living in local communities across the country. The selection of survey households was carried out in a two-stage design. In the first stage, sample areas were extracted by the probability-proportional-to-size sampling, in the second stage, the households were extracted by the systematic sampling (16). A total of 765 trained interviewers (3 interviewers per 255 public health centers) who have received training related to the survey visited the sampled households and conducted one-on-one computer assisted personal interviews (CAPI). The data were collected from August 16 to October 31, 2020, and a total of 229,269 subjects participated in the 2020 KCHS, This study was conducted on 1,071 subjects who had experienced self-quarantine during COVID-19 among 229,269 subjects.

### Study Variables

The following study variables were included, based on George's (15) SAMD:

#### Demographic Characteristics

The demographic characteristics of the study subjects included age, gender, education level, employment, and household income. Age was classified as <40 years, 40–64 years, and >65 years, and education level was classified as elementary school, middle school, high school, and college. Employment was classified as currently employed or unemployed, and household income was classified as <1 million won, 1–2.99 million won, 3–4.99 million won, and >5 million won.

#### Life Event

Life event referred to changes in daily life due to COVID-19. The state of daily life before the COVID-19 pandemic was considered 100 points, complete stoppage of daily life was assigned a score of 0, and no change was given a score of 100 points. Lower scores indicated greater changes in daily life.

#### Vulnerability

Vulnerability was determined by perceived health status, which was measured with the question “How do you usually feel about your health?” The question was scored on a scale ranging from 1 (“very good”) to 5 (“very bad”) points. A higher score indicated worse perceived health status. Perceived health status has good validity as a strong predictor of morbidity, mortality, and use of health care services among various subjects (17, 18) and reported good test–retest reliability (19).

#### Social Integration and Protective Factors

Social integration and protective factors were used to measure the level of family and social support. They included marital status, living arrangement, and whether another person provided assistance during the self-quarantine. Marital status was classified as married or not married (single, divorced, or widowed). Living arrangement was classified as living alone or living with others. The question on whether another person provided assistance during the self-quarantine was answered as “yes” or “no.”

#### Coping

Coping was measured as consultations with psychiatrists for depressive symptoms, which was classified as “yes” or “no.”

#### Depressive Symptoms

Depressive symptoms was measured using the Patient Health Questionnaire-2 (PHQ-2) (20). The PHQ-2 is a self-reporting test to screen for depression, which consists of questions 1 and 2 of the Patient Health Questionnaire-9. Among the diagnostic criteria for major depressive disorder listed in the fourth edition of the Diagnostic and Statistical Manual of Mental Disorders, it consists of two areas: depressed mood and decreased interest, which are core symptoms included in the PHQ-2. Responses are scored on a 4-point Likert scale, ranging from 0 (“not at all”) to 3 (“almost every day”), with the total score ranging from 0 to 6. In this study, a total score of 3 or higher indicated that the subject had depressive symptoms.

### Data Analysis

This study used raw data from the 2020 KCHS, which was a stratified sampling design rather than a simple random sampling design, so it is recommended to apply the complex sampling design for analysis (16). Therefore, we analyzed the data using a complex sample design by applying weights, stratification, and cluster. Descriptive statistical analysis was conducted on the measured variables, and the difference in depressive symptoms according to the measured variables was analyzed using the Rao-Scott X^2^ test. Logistic regression analysis was conducted to identify the factors associated with depressive symptoms. The SPSS/WIN 22.0 program (SPSS, Chicago, Illinois, USA) was used, and the statistical significance level was set to *p* < 0.05.

## Results

### General Characteristics of the Subjects

Of all the subjects, 53.4% were men and 46.6% were women. The average age was 40.01 years. In addition, 63.8% had a college or higher level of education, 42.7% of the household income was 5 million won or more, and 62.7% were employed. The average score for changes in daily life due to COVID-19 was 48.92 out of 100, and the average score for perceived health status was 2.26 out of 5. It was also found that 51.1% were married, and 13.7% lived alone. Eighty-seven of the subjects had someone who could help them during self-quarantine, and 2.1% of the subjects underwent psychiatric counseling for depressive symptoms (Table 1).

**Table 1:** General characteristics of subjects (*N* = 1,071).

**Variable**	**Category**	**Total *n* (%) M ±SE**	**Depressive symptoms**		**Rao-Scott χ^2^(p) /t(p)**
			**No *n* (%) or M ±SE**	**Yes *n* (%) or M ±SE**	
**Demographic factors**				
Age (years)		40.01 ± 0.31	40.31 ± 0.33	34.43 ± 0.73	
	<40	501 (46.8)	465 (92.4)	36 (7.6)	79.59 (<0.001)
	40–64	414 (38.7)	397 (96.9)	17 (3.1)	
	≥ 65	156 (14.5)	152 (99.4)	4 (0.6)	
Gender	Men	572 (53.4)	538 (94.3)	34 (5.7)	0.81 (0.370)
	Women	499 (46.6)	476 (95.0)	23 (5.0)	
Level of education	≤ Middle school	140 (13.1)	132 (94.4)	8 (5.6)	1.29 (0.268)
	High school	247 (23.1)	235 (95.9)	12 (4.1)	
	≥ College	684 (63.8)	647 (94.3)	37 (5.7)	
Household income	<100	86 (8.0)	78 (86.3)	8 (13.7)	11.16 (<0.001)
(unit: KRW 10,000 won/ month)	100–299	248 (23.2)	231 (91.9)	17 (8.1)	
	300–499	280 (26.1)	263 (93.3)	17 (6.7)	
	≥500	445 (42.7)	430 (97.1)	15 (2.9)	
Employment	Yes	671 (62.7)	640 (95.1)	31 (4.9)	1.14 (0.287)
	No	400 (37.3)	374 (94.0)	26 (6.0)	
**Life event**					
Changes in daily life due to COVID-19		48.92 ± 0.52	49.53 ± 0.53	38.12 ± 2.24	4.93 (<0.001)
**Vulnerability factors**		
Perceived health status		2.26 ± 0.02	2.25 ± 0.02	2.60 ± 0.08	−4.14 (<0.001)
**Social integration and protective factors**		
Marital status	Yes	578 (54.0)	557 (96.1)	21 (3.9)	14.52 (<0.001)
	No	493 (46.0)	457 (93.2)	36 (6.8)	
Living arrangement	Living with others	924 (86.3)	878 (95.2)	46 (4.8)	33.43 (<0.001)
	Living alone	147 (13.7)	136 (90.2)	11 (9.8)	
Whether someone provided assistance during the self-quarantine	Yes	932 (87.0)	889 (95.1)	43 (4.9)	8.76 (0.004)
	No	139 (13.0)	125 (91.5)	14 (8.5)	
**Coping**				
Psychiatric counseling due to depressive symptom	Yes	23 (2.1)	16 (68.3)	7 (31.7)	139.39 (<0.001)
	No	1,048 (97.9)	998 (95.1)	50 (4.9)	

There were significant differences in depressive symptoms in terms of age (χ2 = 79.59, *p* < 0.001), household income (χ2 = 11.16, *p* < 0.001), changes in daily life due to COVID-19 (χ2 = 4.93, *p* < 0.001), perceived health status (χ2 = −4.1 4, *p* < 0.001), marital status (χ2 = 14.52, *p* < 0.001), living arrangement (χ2 = 33.43, *p* < 0.001), whether someone provided assistance during the self-quarantine (χ2 = 8.76, *p* < 0.001), and psychiatric counseling for depressive symptoms (χ2 = 139.39, *p* < 0.001) (Table 1).

### Factors Related to Depressive Symptoms

Factors related to depressive symptoms were verified using logistic regression analysis. Being aged 40–64 years [odds ratio (OR) 0.35, 95% confidence interval (CI) = 0.24–0.51], being aged >65 years (OR 0.02, 95% CI = 0.01–0.05), having less than a middle school education (OR 1.98, 95% CI = 1.14–3.44), having a household income <1 million won (OR 5.71, 95% CI = 1.77–18.40), having a household income between 1 and 2.99 million won (OR 2.35, 95% CI = 1.29–4.29), having a household income between 3 and 4.99 million won (OR 2.13, 95% CI = 1.23–3.71), having had changes in daily life due to COVID-19 (OR 0.98, 95% CI = 0.96–0.98), with poor perceived health status (OR 1.49, 95% CI = 1.14–1.93), having not been provided with assistance during the self-quarantine (OR 1.79, 95% CI = 1.13–2.84), and having undergone psychiatric counseling for depressive symptoms (OR 5.00, 95% CI = 2.92–8.57) had statistically significant associated with depressive symptoms (Table 2).

**Table 2:** Logistic regression (*N* = 1,071).

**Variable**	**Category**	**OR (95% CI)**	***p***
**Demographic factor**			
Age (years)	<40	1 (referent)	
	40–64	0.35 (0.24–0.51)	<0.001
	≥65	0.02 (0.01–0.05)	<0.001
Gender	Men	0.88 (0.63–1.22)	0.431
	Women	1 (referent)	
Level of education	≤ Middle school	1.98 (1.14–3.44)	0.016
	High school	0.79 (0.37–1.68)	0.534
	≥College	1 (referent)	
Household income	<100	5.71 (1.77–18.40)	0.004
(unit: KRW 10,000 won/month)	100–299	2.35 (1.29–4.29)	0.006
	300–499	2.13 (1.23–3.71)	0.008
	≥500	1 (referent)	
Employment	Yes	1 (referent)	0.167
	No	1.42(0.86–2.32)	
**Life event**			
Changes in daily life due to COVID-19		0.98 (0.96–0.98)	<0.001
**Vulnerability factors**			
Perceived health status		1.49 (1.14–1.93)	0.003
**Social integration and protective factors**			
Marital status	Yes	1 (referent)	0.175
	No	0.78 (0.54–1.12)	
Living arrangement	Living with others	1 (referent)	0.084
	Living alone	1.58 (0.94–2.67)	
Whether someone provided assistance during the self-quarantine	Yes	1 (referent)	0.014
	No	1.79 (1.13–2.84)	
**Coping**			
Psychiatric counseling due to depressive symptom	Yes	5.00 (2.92–8.57)	<0.001
	No	1 (referent)	

*CI, confidence interval; OR, odds ratio*.

## Discussion

We identified the factors associated with depressive symptoms in individuals who had experienced self-quarantine due to COVID-19 using Lazarus and Folkman's (14) stress, coping, and adaptation theory and George's (15) SAMD.

The prevalence of depressive symptoms identified in this study was higher than that observed in the 2019 KCHS conducted before the COVID-19 pandemic. In a study on adults in the United States, the prevalence of depressive symptoms increased by more than three-fold from 8.5% before the pandemic to 27.8% after the pandemic (12). In another study of 4,335 adults conducted in Germany (6), 31.1% of adults had depression during the pandemic. In addition, 307 (26.5%) out of 1,160 adults had depression during the pandemic in China (11). These results suggest that the COVID-19 pandemic negatively affects mental health. However, the prevalence of depressive symptoms in our study was low compared with other countries, and this may be attributed to the effects of national psychological prevention measures. In Korea, the Ministry of Health and Welfare has formed an integrated psychological support group to provide psychological support such as telephone and face-to-face counseling for the general public, infected individuals and their families, those in self-quarantine, and families of those who died due to COVID-19 infection (21). There is evidence that these measures have lowered the prevalence of depressive symptoms during the pandemic. In addition, according to a report from the Organization for Economic Co-operation and Development, the prevalence of mental health is affected by the strictness of a country's quarantine policies and the number of deaths due to COVID-19 (22). At the time of this study's data collection, the fatality rate due to COVID-19 was 2.03%-3.67% in the United States and Europe, 5.65% in China higher than 1.54% in Korea, which may have affected the prevalence of depression due to COVID-19 (23).

The contextual factors that related to depressive symptoms were age, level of education, and economic status. Consistent with previous findings, younger age and lower education levels were associated with greater depression (6, 11, 24–26). In addition, lower income was associated with higher levels of depression.

This finding corresponds with those of previous studies that indicated that financial problems cause serious socioeconomic distress and increase depression (2, 11, 12, 21, 26). Brooks et al. (2) reported that individuals with low incomes are more likely to be affected by temporary income loss during self-quarantine than those with high incomes. Therefore, if possible, financial compensation should be provided to individuals with low income who self-quarantine, and policies should be developed to provide such compensation.

Our findings showed that changes in daily life due to COVID-19 had related to depressive symptoms, with greater changes in daily life being associated with higher depressive symptoms. Similar findings were observed in previous studies (6, 25, 27) in which changes in daily life, such as social distancing, working from home, delayed first day of school, and difficulties in using hospitals due to COVID-19, may lead to various psychological problems such as personal stress, anxiety, depression, fear, anger, and loneliness. Also, in a qualitative study examining the experiences of the older adults about the changes in their daily life due to COVID-19, similar findings were observed which complained of boredom, isolation, depression and anxiety while experiencing limited use of welfare centers for the elderly and job interruption (28). In particular, changes in daily life can lead to conflicts in various relationships. Increased time spent at home due to the COVID-19 pandemic has led to more family conflicts (21), and at school and work, conflicts in interpersonal relationships over prevention measures such as wearing masks have increased (29). Such mistrust and conflicts in relationships can lead to secondary traumatic experiences and severe depression (30–33). Therefore, to minimize the changes in daily life due to COVID-19 and to aid individuals in adapting and coping with new daily lifestyles, active countermeasures must be sought. Moreover, efforts are required to reduce the conflicts that may occur in various relationships.

We observed that the presence of someone who could help during self-quarantine was a protective factor against depressive symptoms. In this study, social support did not refer to the level of actual help, but perceived support that subjects could rely on someone for help when needed. This suggests that the perception of a social network rather than the actual exchange of social relationships may help alleviate depression (34). Previous studies showed that social support has positive effects, such as reducing depression through the actual exchange of resources (9, 35, 36), and based on these findings, measures focusing on offline-centered direct interactions through expansion of social networks have been mainly suggested. However, quarantine measures on social distancing limit the active implementation of such strategies. Therefore, our findings on the effects of perceived support may be significant for the reduction of depressive symptoms during the COVID-19 pandemic, in which prevention is heavily focused on social distancing. Perceived social support and mutual trust are strong protective factors for mental health and act as universal psychological safety nets (37). Thus, during the COVID-19 pandemic, which limits direct interactions between individuals, it is important to maintain a positive psychological bond with friends and neighbors using various resources, such as active communication by phone, e-mail, and social network services. In a qualitative study of college students' experiences of daily life changes due to COVID-19, psychological bonding was expressed as “the aesthetics of triviality,” he said that when he was surrounded by feelings of isolation, the other person sensitively grasped it, paid attention to it, and was grateful for a simple call asking for his/her best regards (38). Such psychological bonding promotes emotional stability and self-esteem for psychosocial adaptation and enhances problem-solving ability, thereby having positive effects on mental health (39).

In our study, poor perceived health status was a vulnerability factor, leading to greater depressive symptoms. This finding is consistent with those of previous studies (9, 40). Perceived health status is more closely related to depressive symptoms than chronic disease and functional status, which are objective indicators of physical health. Therefore, to help promote positive perceived health status, measures such as online health promotion, physical exercise, and health education programs are necessary during the COVID-19 pandemic.

Our data showed that depressive symptoms were higher in those who received psychiatric counseling for depression. In agreement with our findings, a previous study showed that experiences of counseling or treatment for depression are a behavioral coping style to overcome depressive symptoms and that more experiences of counseling or treatment lead to more depression (41). This means that the experience of treatment for depression is a positive coping behavior to overcome depression, and at the same time, it is a risk factor for exposure to depression or a risk of recurrence (40). Therefore, further in-depth studies should be conducted on the relationship between the experiences of depression treatment and depressive symptoms.

### Implications for Public Health

We systematically and comprehensively identified the factors associated with depressive symptoms based on Lazarus and Folkman's (14) stress, coping, and adaptation theory and George's (15) SAMD. Among contextual factors, age, level of education, and economic status were factors related to depressive symptoms, suggesting that policies on COVID-19 measures should consider the characteristics of subjects. In addition, this study is significant as it identified the vulnerability and protective factors of depressive symptoms and provided basic data for the development of programs to alleviate and prevent depressive symptoms in self-quarantine individuals.

As the COVID-19 pandemic continues, long-term measures such as vaccination are being encouraged and efforts such as “With Corona” are being carried out to return to pre-COVID-19 pandemic daily life. However, self-quarantine remains an important preventive measure against the spread of the disease. Therefore, mental health should be a primary concern during self-quarantine. Systems to screen for those with vulnerable mental health before self-quarantine should be established and implemented, and mental health assessments should be regularly conducted even during self-quarantine. Thus, if a high-risk group or a person with symptoms related to mental health is found, active psychological support, such as referral to specialized mental health services, should be provided. In addition, systems to follow up and manage mental health after self-quarantine should be prepared as well, and various psychological support services should be developed to prevent the onset of mental health problems such as depression at an early stage and mental health should not deteriorate through continuous monitoring.

### Limitations

Our study has some limitations. First, we used cross-sectional data, there is a limitation in that it is difficult to accurately identify a causal relationship. Second, the PHQ-2 used in this study has limitations as it is a screening tool, not a diagnostic tool. Third, in this study, only data on depression symptoms that occurred over the past 2-weeks were collected and used. Although the period between the end of self-quarantine and the time of the survey was not clearly known, the symptoms of depression after self-quarantine were investigated. Nevertheless, we could not exclude subjects who might have previously depressive symptoms. Fourth, we assessed daily life changes with a single item. However, the validity and reliability of the single item have not been reported in previous studies. In the future, it is suggested to verify the reliability and validity of the scale. Finally, we did not measure various coping strategies (e.g., use of medication, psychotherapy, and locus of control) that were proposed in SAMD (15). In the future, it is suggested to conduct research including various coping strategy variables.

## Data Availability Statement

The data that support the findings of this study are available from the corresponding author, upon reasonable request.

## Ethics Statement

The studies involving human participants were reviewed and approved by an Institutional Review Board of the Gachon University (No. 1044396-202109-HR-198-01). The patients/participants provided their written informed consent to participate in this study.

## Author Contributions

H-YJ and YK conceived and designed the study and analyzed the data. H-YJ and S-YH wrote the first draft. All authors contributed to revisions of the manuscript and critical discussion and have read and agreed to the published version of the manuscript.

## Conflict of Interest

The authors declare that the research was conducted in the absence of any commercial or financial relationships that could be construed as a potential conflict of interest.

## Publisher's Note

All claims expressed in this article are solely those of the authors and do not necessarily represent those of their affiliated organizations, or those of the publisher, the editors and the reviewers. Any product that may be evaluated in this article, or claim that may be made by its manufacturer, is not guaranteed or endorsed by the publisher.
